# Annotations

**Published:** 1892-01-02

**Authors:** 


### ANNOTATIONS.

**London's Foggy Christmas.**

CHRISTMAS Day was the sixth day of almost unmitigated fog, and it was one of the worst days of the six. Almost all the newspapers are beginning to take up with energy the subject of foggy London. We are not entitled to boast ourselves of our science until it has been reduced to practice. A railway on paper would have been little better than a flying machine; it is the railway on the lines and with comfortable carriages and an active engine in front that is really serviceable. We are not indisposed to admire scientific men in their laboratories; and we pay them the very great compliment of usually taking them at their word. But science is like all other knowledge, a very barren business, until it is brought down to working facts. The Thames valley is naturally a foggy region, and would be foggy even if there were no London in it. So much must be admitted; and it is possible that the natural fogginess of the valley cannot easily be cured. But it could at least be endured if the fog consisted of watery vapour pure and simple. It is what we throw into it from our house, factory, and office chimneys that makes the fog of London so black, so depressing, so deadly. It is not necessary to describe that with which we are all so familiar. What is wanted is a method of cure. It will be imperative first and chiefly to put an end to the production of smoke. Heat and light without smoke are the things to be aimed at. The thing can be done; it is not even difficult in the laboratory. It is only in the reduction of science to practice that difficulty comes in. But the problem will have to be faced and dealt with. London is not habitable during a black fog any more than a battle field is a place of safety during a fight. The business men of London who find their fortunes in its warehouses, its offices, and its shops will have to take the subject to heart; the shopkeepers especially must consider the question. If they do not, their business during the winter will be ruined. Doctors know very well the terrible dangers that lurk in London fogs; and their patients are learning to know quite as well. Already there is a Christmas exodus from the metropolis which seriously diminishes the profits of business. But the exodus will become more and more decided, and more and more general as people learn to properly value the damage done to health and life by successive fogs. Londoners should insist upon their County Council's immediate and practical consideration of the subject. Money would solve the problem. No doubt the sum required to convert London into an electrically heated and lighted city would be very great; but it would be the price of comfort, health, and financial prosperity. At any rate, there is no doubt of this that if the fogs continue, and continue to increase, nobody will live in London who can afford to live out of it. It will become a city entirely of offices, warehouses, and poor people. The prospect is not encouraging, especially to those who carry on costly retail businesses in the City, the West-end, and the wealthy suburbs.

**The Influenza.**

THERE appears to be some reason to hope that London may to a large extent escape the epidemic of influenza which has been for some time prevalent in England. The outbreak, which began in Cornwall at the beginning of the winter period, has now reached Scotland, taking in its journey north Exeter, Bristol, Staffordshire, Newcastle-on-Tyne, Berwick, Edinburgh, Dundee, and the Orkneys. Up to the present time London, Manchester, Sheffield, Leeds, and Hull, all of which suffered severely either at the beginning of the present year or during 1890, have to a large extent escaped. It is too early to speak with any very great confidence when we remember that in 1890 the epidemic did not reach its maximum until January. Still, as we have now had a long period of wet and most depressing weather in London without any marked ill effects, we may at any rate begin to breathe a little more freely. The death-rate in Edinburgh and some other towns has been markedly affected by the epidemic. During the week ending December 5th the mortality was almost double that of the normal rate. There was a total of one hundred and eighty-five deaths, equal to a death-rate of thirty-seven per thousand of the population. Of these no fewer than one hundred and nineteen persons died of chest diseases, such as pneumonia, pleurisy, bronchitis, and so on. Some few seem to have sunk from sheer exhaustion. On the whole, however, the epidemic has been moderate, both in its extent and in its severity, as compared with those of the last and the previous winters. The weather in London, as everybody knows, has been phenomenally mild and wet, "disgustingly mild," as somebody has called it. Happily, according to the doctrine of averages, it must soon cease, and the sooner the better; an outbreak of influenza in London would be a most inopportune visitation.

**American Physicians on Virchow.**

THE Johns Hopkins University at Baltimore publishes a monthly circular, giving an account of any work of general interest which may have been done within the month. The November circular records the celebration at the University of Professor Virchow's seventieth birthday. The President and medical professors issued invitations to the University staff and leading physicians of Baltimore; and a distinguished company met in response to the invitations in the lecture room of the physical laboratory. Professor Ira Remsen, M.D., Ph.D., was called to the chair. All nations still continue to indulge in hero-worship. America is no exception. It is encouraging to find that the heroes of other nations are sought out for admiration, as well as those of American nationality and blood. Science may have a great mission to perform in teaching the identity of all branches of the human family, and in bringing that practically important doctrine into general recognition. There are those who continue to believe in the purifying and beneficient influences of pestilence and war. Science is the steady enemy of both those methods of devastating humanity. War is the stupidest and most unsatisfactory of all the possible ways of settling differences; pestilence is the dread fruit of ignorance, practical incompetence, and vice. Science would substitute reason for the sword, and knowledge and effective capacity for the ignorance which grows to pestilence. Prof. Remsen eulogises Virchow because he has not permitted himself to be confined to a narrow specialty. He has been and is a worker in that department of medicine called pathology, and therein has distinguished himself above all his contemporaries. But he is also an anthropologist and an archæologist. He has worked at the science of man: man physical, and man mental and moral: man as he presents himself to the physiologist to-day, and man as he has presented himself to the historian and the philosopher in all ages. Moreover, Virchow is and has long been that *rara avis*, a medical politician. We have a few physicians in this country who follow politics; and not one of them has succeeded in distinguishing himself in the public service. But Virchow has shown a fine aptitude for everything he has put his hand to. It was Carlyle who said he did not believe that a man was a great man at all unless he could be great in everything to which he devoted his full attention. Virchow has fulfilled these very exacting conditions of great manhood. The physicians of the Johns Hopkins University, in honouring Virchow with such spontaneous warmth have honoured themselves in the eyes of the world. When we are called upon to celebrate the marriage of science with a reasonable religion, as we shall be soon, we may begin to look for the dawn of that golden age which poets have placed in the past, but which men of more philosophic temperament anticipate in the brightening future.

